# In cats which treatment, meloxicam or prednisolone, most quickly reduces clinical signs of feline interstitial cystitis?

**DOI:** 10.18849/ve.v7i1.346

**Published:** 2022-01-26

**Authors:** Thomas Smith-Uchotski+

**Keywords:** BLADDER DISEASE, CAT, FELINE IDIOPATHIC CYSTITIS, FELINE INTERSITIAL CYSTITIS, FELINE LOWER URINARY TRACT DISEASE, MELOXICAM, PREDNISOLONE

## Abstract

PICO question

In cats with feline interstitial cystitis, which therapy brings a faster resolution of clinical signs: meloxicam or prednisolone?

Clinical bottom line

Category of research question

Treatment

The number and type of study designs reviewed

Two papers evaluated as relevant to the PICO question were critically reviewed. Both were double-blinded randomised controlled trials.

One paper not related to the PICO question, a single-blinded randomised controlled trial, was also reviewed as it is touched upon in the discussion section

Strength of evidence

Appraisal of the literature reveals weak evidence that meloxicam and prednisolone are of equivalent effectiveness when treating feline interstitial cystitis, also known as feline idiopathic cystitis (FIC)

Outcomes reported

There is no statistically significant difference in the reduction of clinical signs when meloxicam is compared with a placebo for the treatment of FIC. There is no statistically significant difference in reduction of clinical signs when prednisolone is compared with a placebo for the treatment of FIC. No studies were available for review which directly compared meloxicam against prednisolone as treatment options for FIC

Conclusion

In cats with FIC, insufficient evidence exists to truly conclude whether meloxicam or prednisolone is the most efficacious therapy for the reduction of clinical signs. Two double-blinded randomised controlled trials were evaluated – one compared the efficacy of meloxicam against a placebo; the other compared the efficacy of prednisolone against a placebo. Neither study found a statistically significant difference between the assessed treatment modality and the placebo used in reducing the clinical signs of FIC. As such, weak evidence exists that there is no significant difference between the use of meloxicam and a placebo, and prednisolone and a placebo in the reduction of clinical signs of FIC. Additionally, it could therefore be hypothesised that no significant difference exists in the reduction of clinical signs when comparing meloxicam against prednisolone as treatments for FIC however, no study was discoverable which was able to substantiate this claim

## How to apply this evidence in practice

The application of evidence into practice should take into account multiple factors, not limited to: individual clinical expertise, patient’s circumstances and owners’ values, country, location or clinic where you work, the individual case in front of you, the availability of therapies and resources.

Knowledge Summaries are a resource to help reinforce or inform decision making. They do not override the responsibility or judgement of the practitioner to do what is best for the animal in their care.

## Clinical scenario

Feline interstitial cystitis (FIC), also known as feline idiopathic cystitis and historically as idiopathic feline lower urinary tract disease (FLUTD), is one of the most commonly diagnosed lower urinary tract diseases of cats. As a clinician, you are aware that the clinical signs brought about by the condition are caused by an inflammatory reaction. Commonly, anti-inflammatory treatments are a staple of treatment for this condition. Both meloxicam and prednisolone offer anti-inflammatory action; you wonder which is the most efficacious treatment in reducing the clinical signs of FIC.

## The evidence

Unfortunately, no studies directly comparing the effectiveness of meloxicam against prednisolone as treatments of FIC were uncovered. Two studies which partially answered the PICO question were uncovered. One was a double-blinded randomised controlled trial comparing meloxicam against a placebo (Dorsch et al., 2016). The other was a double-blinded randomised controlled trial comparing prednisolone against a placebo (Osborne et al., 1996). Two studies were omitted from assessment due to being inaccessible.

The first paper, by Dorsch et al. (2016), comparing meloxicam against a placebo, concluded that there was no statistical significance in the reduction of clinical signs when comparing the two study groups. While the number of participants in the study was relatively substantial (n = 37), no power calculations were performed to assess if the number of participants was sufficient to provide viable evidence. Additionally, the responsibility of data recording was passed from the researchers to animal owners, which could introduce a high degree of variability in the quality of data recording, potentially skewing the data.

The second paper, by Osborne et al. (1996), comparing prednisolone against a placebo, also concluded that there was no statistical significance in the reduction of clinical signs when comparing the two study groups.

## Summary of the evidence

**Dorsch et al. (2016):** 

Population:	Cats exclusively with obstructive feline interstitial cystitis (FIC). Population selection: Cats presenting with signs of feline lower urinary tract disease (FLUTD) (leading to a diagnosis of FIC) were submitted. The following criteria were then applied: Animals had to have obstructive FIC (unsuccessful voiding with a painful, distended bladder on abdominal palpation). Cats with evidence of urolithiasis or neoplasia were excluded. Those with concurrent disease (e.g. hyperthyroidism, diabetes mellitus) were excluded. Cats treated with NSAIDs, antimicrobials or steroids up to 2 weeks before presentation were excluded. Cats were withdrawn from the study if urine cultures did not return negative at any point throughout the study.
Sample size:	37 cats with obstructive FIC, separated into two groups: Group 1: Cats treated with meloxicam (n = 18). Group 2: Cats treated with a placebo (n = 19).
Intervention details:	37 cats were admitted to a veterinary hospital and had the following procedures performed: Intravenous fluid therapy with lactated Ringer’s solution upon admission for 48 hours. Intramural catheter placement upon admission. This was removed after 48 hours. Treatment with subcutaneous buprenorphine (0.01 mg/kg) q8h for 48 hours. Urinalysis was performed on admission, repeated on days 2 or 3 of hospitalisation, and again on a re-check examination 10 to 14 days after discharge. 24 hours after admission, intervention was initiated using either oral meloxicam or an oral placebo once daily. Doses administered: Group 1: Day 1: 0.1 mg/kg of meloxicam. Days 2–5: 0.05 mg/kg of meloxicam. Group 2: Days 1–5: Placebo dose (quantity undefined by study). Animals were discharged from the hospital and owners were given written instructions on how to complete the 5-day treatment course at home, following the treatment protocol required for the study. Recording of results: While in the hospital, daily physical examinations were performed to measure the desired parameters (outlined below). The results of the urinalyses (on day 0, 2 or 3, and at the post-operative check) were also recorded and evaluated. Upon leaving the hospital, owners were given a standardised questionnaire to answer daily about their cat's general condition, voiding behaviour and food intake.
Study design:	Double-blinded randomised controlled trial.
Outcome studied:	Main outcome assessed: Time to developing recurrent urethral obstruction. Variables measured: In hospital: General demeanour. Pain on abdominal palpation. Food intake. Urinalysis results: Protein. Red blood cells per high power field. White blood cells per high power field. Urine specific gravity. Owner questionnaire: General demeanour. Food intake. Voiding behaviour (pain, stranguria, pollakiuria, periuria).
Main Findings (relevant to PICO question)	No statistically significant difference was found in the time to reduction of clinical signs between the use of meloxicam and the use of a placebo (no intervention) in cats with obstructive FIC. P-values were calculated comparing the placebo group to the meloxicam group in a pairwise fashion for each day that data were collected. During the hospitalisation period (days 0 to 3) and at recheck, P-values, which compared group 1 against group 2, ranged from 0.235 to 1.000, or were quoted as being non-applicable for measures of general demeanour, pain on abdominal palpation and food intake. The remainder of in-hospital measurements taken were quoted as having a P-value of 'NS' – not significant. On day 0 of hospitalisation, macroscopic haematuria compared between the two groups was shown to have a statistically significant P-value of 0.049. (However, treatment had not been initiated at this point in time, so this only shows a difference in the populations at the study's outset). Comparisons of urinalysis results showed no statistically significant difference between the placebo and meloxicam groups. (P-values were quoted as 'NS', not significant, or 'NA', not applicable). No statistically significant difference was seen when comparing the two groups for any measure made on the owner questionnaire at home. All P-values were quoted as 'NS' – not significant. Persistence of clinical signs in excess of 7 days in most of the study animals could show that symptomatic treatment of FIC is required for a prolonged period of time.
Limitations:	Initial intervention (with meloxicam and the placebo groups) overlapped administration of subcutaneous buprenorphine on the first day of administering oral medication. This could potentially confound the results for day 1 of the trial. A lot of different assessors were involved in data recording – potentially up to 37 owners as well as the involved study leads. This could cause variation in the recordings, especially with respect to subjective measurements. The recording duties per individual animal were passed from clinicians to owners, which could also cause variation in recordings, as well as throw into question the reliability of medication dosing at home. Specific details about the volume of placebo administered and the ingredients used were not included. Use of an oral intervention while animals could potentially have reduced food intake could lead to inaccurate dosing of meloxicam and the placebo. No power calculations were performed.

**Osborne et al. (1996):** 

Population:	Cats exclusively with idiopathic feline lower urinary tract disease (FLUTD). Population selection: Diagnosis of idiopathic FLUTD was made on the following grounds: History and clinical examination were consistent with idiopathic FLUTD. Urinalysis and quantitative culture of urine for aerobic and mycoplasma (examining for a negative culture). Plain and contrast radiography (intravenous urography followed by antegrate cytrourethrography) of the urinary tract to show no radiodense uroliths. Complete blood cell counts. Serum chemistry profiles (all major enzymes and electrolytes) were within normal limits. Feline leukaemia antigen tests were negative.
Sample size:	11 individual cats, one of which entered the study twice due to having a second episode of idiopathic FLUTD 6 months after finishing its initial trial, were entered into the study (n = 12). These animals were split into two groups: Prednisolone: Cats treated with prednisolone (n = 6*). Placebo: Cats treated with a placebo (n = 6*). *The cat which entered the trial twice was initially treated with prednisolone. Upon re-entry to the study 6 months later, it was treated with a placebo. All capsules were gelatin capsules.
Intervention details:	11 cats with idiopathic FLUTD were identified after being screened using the aforementioned criteria. Upon admission to the study, urine samples were collected either by cystocentesis (n = 7) or by voluntary voiding (n = 5). The cats were split into two groups, an intervention group treated with prednisolone and a control group treated with a placebo: Prednisolone**: **0 mg/kg prednisolone per os, BID, in capsules (n = 6). Placebo**:** Cats treated with a placebo in capsules (n = 6). The placebo was sealed in capsules identical to that which the prednisolone group received. Both groups were hospitalised for, and received their respective interventions, for 10 days. All animals were fed Science Diet Feline maintenance, Hill's Pet Nutrition, for the duration of the study. While hospitalised, the remaining urine samples were collected via voiding to prevent confounding data from iatrogenic haematuria caused by cystocentesis and catheterisation for contrast radiographs. All urine samples were analysed within 30 minutes of collection or refrigerated and analysed within 1 to 12 hours. During the hospitalisation period, urine was collected and analysed daily. Clinical signs were also assessed. After discontinuation of treatment, urinalysis and bacterial cultures were performed on days 4 and 18 after stopping therapy (2 and 4 weeks after starting the study). These samples were collected by cystocentesis.
Study design:	Double-blinded randomised controlled trial.
Outcome studied:	Main outcomes assessed: Time to reduction of clinical signs. Variables measured: Urine Dipstix: pH. Glucose. Acetone. Bilirubin. Protein. Occult blood. Urine sediment exam: Presence of crystalluria. Time to remission of microscopic haematuria. Clinical examination: Time to resolution of clinical signs (dysuria and occult haematuria are specifically noted). Radiography* (plain and contrast studies): Presence of radiodense uroliths. *This criterion was only used in the initial screening process and was unrepeated throughout the study. It has been included here for completeness.
Main Findings (relevant to PICO question)	No significant difference was found in the time to reduction of clinical signs between the use of prednisolone and the use of a placebo (no intervention) in cats with idiopathic FLUTD.
Limitations:	Cats in the hospital had daily urinalyses. Only days 0, 5 and 10 are published. Therefore, we do not know if these omitted days contained results which may influence the outcome of the study. One cat was re-submitted to the study. While the cat was put through both the treatment and control group, potentially furthering the evidence that there is no significant difference between a placebo and prednisolone this could skew the data as this patient's individual factors have been entered into the study multiple times. No justification was given as to why some cats were initially sampled using cystocentesis, where there is a risk of iatrogenically causing haematuria, as all cats at the start of the study were not blocked so could void naturally. Once released from hospital the cats may hunt, which could confound data, which was originally controlled for by feeding the cats a uniform diet when in the hospital. Urine samples were not all stored in a consistent manner. Some were analysed within 30 minutes of animals voiding, whereas others were analysed post-refrigeration after 1 to 12 hours, which could introduce artifactual change into the results. The study mentions that urine specific gravity was analysed but not by what means. Both Dipstix and refractometry can offer a USG result, where they deliver a subjective and objective measurement respectively. If measured by Dipstix, then the USGs may be subject to personal interpretation, which could skew the results if multiple assessors were used to interpret the tests.. No power calculations were performed. No P-values or confidence intervals were calculated for this study, making it difficult to truly appreciate if there was a true statistically significant difference or not.

**Nivy et al. (2019):** 

Population:	Male cats with obstructive feline interstitial cystitis (FIC). Population selection: No cat had received medication before admission to the hospital. Resolution of azotaemia and the ability to fully empty the urinary bladder before discharge from the hospital. Obstructive FIC diagnosed on the basis of compatible history, clinical signs, and physical examination findings (stranguria, haematuria, pollakiuria, periuria, unsuccessful voiding attempts, and presence of a large, tense painful urinary bladder unamenable to manual voiding). Abdominal ultrasonography, urinalysis and urine cultures with results compatible with FIC. 20/51 cats also had survey radiographs and contrast retrograde urethrography performed on them for diagnosis of FIC. (The remaining 31 did not due to financial constraints of the owners). Additional inclusion criteria: Resolution of azotaemia prior to discharge from the hospital and the ability to fully empty the urinary bladder.
Sample size:	Male cats with obstructive FIC (n = 51, of which 7 were intact and 44 were neutered). Intervention group: n = 24*.* Control group: n = 27.
Intervention details:	All cats were admitted to a veterinary hospital. After an initial diagnosis of FIC and enrolment to the study, cats were screened using the aforementioned criteria in the population selection section. Following this, the patients were stabilised in the hospital and obstructions in each cat were relieved using catheterisation. The size of catheter selected was at the clinical judgment of the overseeing clinician. Additional therapies were also delivered at the discretion of the overseeing veterinarian, including fluid choices (rate and type), treatments for hyperkalaemia (insulin and/or bicarbonate) and administration of intravenous dextrose. Timing of the catheter removal was also determined by the overseeing veterinarian’s clinical judgment. In general, these were removed when azotaemia had resolved on blood work and the urine turbidity appeared normal. Following discharge from the hospital after a 3–5-day period (after resolution of clinical signs), the cats were prescribed a therapeutic, urinary dry food diet and allotted to one of two treatment groups: Intervention Group: Alprazolam (0.125 mg/cat, q12h), phenoxybenzamine (2 mg/cat, q12h) and meloxicam (0.025 mg/kg, q24h). Control Group: Alprazolam (0.125 mg/cat, q12h) and phenoxybenzamine (2 mg/cat, q12h). NB: All medications were delivered per os. Owners were also instructed to introduce husbandry changes to their animals including environmental enrichment and stress reduction. Recording of results: Data regarding the individual animals were extracted from the medical records for statistical analysis. These parameters included the signalment, season, number of episodes of FIC signs, environment, diet prior to hospitalisation and after discharge, clinical signs (and duration), vital signs on admission, other therapies given (e.g. antibiotic treatment), weight, and the duration and type of urinary catheter used. Cats were followed at 10 days, 1 month, 2months and 6 months after discharge where they were evaluated for recurrence of FIC related signs and/or signs of a recurrence of urethral obstruction.
Study design:	Single-blinded randomised controlled trial.
Outcome studied:	Time to recurrence of urethral obstruction.
Main Findings (relevant to PICO question)	None – time to recurrence of urethral obstruction was the outcome measured, not time to reduction of clinical signs. Recurrence of obstruction was not found to be statistically significant at any subsequently measured time frame. Odds ratios showed the following relationship between the two groups: <10 days: P = 0.47, 95% CI [0.96, 1.13]. 10 days to 1 month: P = 0.99, 95% CI [0.89, 1.04]. 1–2 months: P = 0.49, 95% CI [0.83, 1.03]. 2–6 months: P = 0.99, 95% CI [0.14, 8.13]. Cumulative recurrence of obstruction was also found to not have any statistically significant measures. Odds ratios comparing the two groups provided P-values and confidence intervals for recurrence within: 1 month: P = 0.99, 95% CI [0.07, 19.12]. 2 months: P = 0.61, 95% CI [0.03, 3.59]. 6 months: P = 0.70, 95% CI [0.13, 2.97]. Recurrence of clinical signs without obstruction within 6 months was also not statistically significant (P = 0.34, 95% CI [0.33, 36.67]), as calculated by odds ratios comparing both groups. This reference has been analysed in detail for completeness as it is mentioned heavily in the appraisal, application and reflection section.
Limitations:	Several cats were treated with antibiotics despite not being in-line with current therapy recommendations for FIC treatment. It is unclear which individuals were treated. While a P value found no significant difference between those treated and those not treated with antimicrobials, this could still be a confounding factor. There was no study group which used a placebo as a control. Therefore, it is difficult to determine if alprazolam and phenoxybenzamine alone are sufficient to reduce recurrence rates. If they are, and meloxicam is not, then we would see no significant difference between the control and study group. However, the possibility exists that we may see a difference between the present control group and a group treated with the placebo. 025 mg/kg q24h of meloxicam may be too low a dose to be of clinical benefit. A paper by Carroll et al. (2011), found that 0.05 mg/kg q24h was the lowest efficacious dose of meloxicam for pain relief when treating sodium urate-induced synovitis in cats. The study only has a power rating of 80% from power calculations, which is slightly underpowered to detect small differences. A lot of treatments were carried out by the owners at home, which could introduce variability and unreliability in the way each animal was handled and could confound results. Many owners did not stick to the desired prescription diets, with some returning to non-therapeutic, standard diets. This could confound results. It is never overtly stated which therapeutic diet (or diets) the owners are asked to obtain. From the paper it could be assumed that either or both of Hill's Prescription Diet c/d Multicare or Royal Canin Urinary Care were recommended; however, it would still be better for all animals to be on the same diet. The study took place over a period from 2016–2018. Different clinicians may have been involved with the study, thereby introducing further variability in recording of the results.

## Appraisal, application and reflection

The most relevant, available evidence excavated were two double-blinded randomised controlled trials. Both explored the time to reduction of clinical signs of cats with feline interstitial cystitis (FIC). One study evaluated the efficacy of meloxicam as a treatment when contrasted with a placebo (Dorsch et al., 2016), where the other evaluated the efficacy of prednisolone as a treatment when contrasted against a placebo (Osborne et al., 1996). Both studies only partially answered the PICO question, as neither directly contrasted meloxicam against prednisolone as therapy modalities.

The study by Dorsch et al., 2016, concluded that there was no statistical significance between the use of meloxicam and the use of a placebo as treatments for FIC, as well as the likelihood of recurrent urethral obstruction (rUO). The main outcome of the study was the time to development of rUO, with the clinical signs being used as indicative measures for when this occurred. The cats in this study all were obstructed at the start of the study (Dorsch et al., 2016).

Superficially, as a double-blinded randomised controlled trial, the study by Dorsch et al., 2016, stands to potentially provide good clinical evidence. While randomised controlled trials are traditionally regarded as strong evidence, the experimental design is the true factor which determines how reliable the evidence generated is (RCVS Knowledge, 2015). Unfortunately, several factors in the study design potentially weaken the study’s overall evidential strength.

All animals were initially treated with buprenorphine (in addition to meloxicam or the placebo). The effects of buprenorphine, in addition to the meloxicam or placebo, were not part of the intended study outcome and as a consequence, overlap of the treatments with buprenorphine and meloxicam (or the placebo) could confound the data which was gathered for day 1 of treatment with meloxicam and/or the placebo. Additionally, recording of data was passed from hospital assessors to a multitude of up to 37 different owners. Owners were expected to assess their animals via a survey devised by the researchers, which removes all of the objective parameters measured in the hospital such as heart rate and respiration rate. As the survey omits many of the previously measured parameters and is purely subjective with up to 37 different and potentially untrained assessors, this could introduce a lot of variability in the data recorded during this portion of the study.

The study quoted a P-value of 0.049 when comparing day 0’s results for macroscopic haematuria when comparing the meloxicam group against the placebo group, implying a statistically significant difference between the two groups. As one group had statistically significantly worse clinical signs than the other, it could mean that the meloxicam group was more severely affected than the other, which could have affected the time to resolution of clinical signs, being in a more progressed state from the outset. However, as no interventions had been administered at the time of this measurement, this is potentially an irrelevant observation when comparing meloxicam to a placebo, since neither therapy had been initiated. Additionally, P-value calculations for each subsequent day showed no significant difference between the two groups following the admission of both interventions, so it could still be concluded that there is no statistically significant difference between either protocol.

The double-blinded randomised controlled study by Osborne et al. (1996), compared the efficacy of prednisolone against a placebo in reducing the clinical signs caused by idiopathic FLUTD. Similarly, this study also found that there was no statistically significant difference between the different treatment modalities. The main outcome of this study was the time to reduction of clinical signs (Osborne et al., 1996).

Similarly to the study by Dorsch et al. (2016), the study design also stands to potentially provide strong evidence as a double-blinded randomised controlled trial. However, there are also factors of this study's individual design which cast some shade over the strength of the study. The study sometimes proves to be a little sparse with its reporting of data; despite urine samples being taken daily from animals, only those from days 0, 5 and 10 are included in the study. Additionally, it is not explicitly mentioned how urine specific gravity is measured. These omissions weaken the overall trust in the study, especially as urine specific gravity has both subjective (Dipstix) and objective (refractometry) measures of being assessed. Additionally, the sample size of the study is very small, where n = 12, and as such the study group may be too small to provide sufficient power. Lastly, no calculations to quantify statistical significance were quoted, which throws doubt over whether the evidence provided actually shows statistical significance or not.

One other single-blinded randomised controlled trial by Nivy et al. (2019), assessed the recurrence rates of FIC and rUO in cats. While this study did not answer the PICO question, the study itself does provide some interesting food for thought. This study compared the use of phenoxybenzamine and alprazolam with or without low dose meloxicam (0.025 mg/kg, q24h, PO) as a treatment for cats with FIC. 51 males with urethral obstruction and FIC were allocated one of the treatment protocols in a single-blind fashion. The study drew the conclusion that the addition of low-dose meloxicam to phenoxybenzamine and alprazolam treatment did not make a significant difference in the return to urethral obstruction and/or FIC compared with cats only treated with phenoxybenzamine and alprazolam (Nivy, et al., 2019).

While the reduction in clinical signs was not measured in this study, it is interesting to note that the addition of meloxicam did not provide any clinical benefit for the assessed parameters. Another study, examining cats with experimentally induced synovitis, showed that low-dose meloxicam of 0.025 mg/kg q24h PO did not make a significant in the reduction of pain signs in comparison to a placebo. However, higher dose rates of 0.05 mg/kg and 0.075 mg/kg with the same dosing interval showed a statistically significant reduction in pain parameters (Carroll et al., 2011). It could therefore be possible that the dose rate chosen by Nivy et al. (2019), was not high enough to be efficacious.

Taking into account all papers cited, it is key to note that the populations of animals studied are slightly different. Both studies which used meloxicam in their treatment protocols – Dorsch et al. (2016), and Nivy et al. (2019), – used cats with obstructive FIC as their populations. By comparison, the paper by Osborne et al. (1996) used animals with non-obstructive FIC as their study population. While all cats had FIC by definition, those with obstructive FIC present with a much more severe clinical syndrome than those with non-obstructive FIC, which raises the question as to whether these populations are truly comparable. As such, it may be good to be more specific with the population and contrast animals exclusively with non-obstructive or obstructive FIC. That being said, it may only be possible to compare these therapies in non-obstructed animals as prednisolone is often contraindicated when an indwelling catheter is present (Osborne et al., 1984).

One other thing to note as well is that steroids and NSAIDs are not the only treatments which are available for treatment of FIC in cats. Many other treatment modalities including amitriptyline, tolfenamic acid, polysulphated glycosaminoglycans and pentosan polysulphate which were not explored in this Knowledge Summary are available and may be worth further consideration and evaluation in another Knowledge Summary. One paper noted that ketoprofen was often the choice NSAID for use in Canada for managing pain associated with musculoskeletal disease, and is often used in treatment of FIC (Dowling, 2000).

In summary, no studies directly comparing the efficacy of meloxicam against prednisolone as a means of reducing the clinical signs of cats with FIC could be identified. At an even more specific level, no studies exist comparing these treatments in populations of cats exclusively with non-obstructive or obstructive FIC. The studies uncovered, all of which were randomised controlled trials, had flaws in their experimental design or, possessed sufficient limitations which weaken their strength as valid evidence. It could be hypothesised that, since both relevant studies found no significant difference between the use of a placebo or a treatment (either meloxicam or prednisolone), there would be no significant difference between using prednisolone and meloxicam. However, the study examining meloxicam used only cats which were obstructed (Dorsch et al., 2016) whereas the study examining prednisolone only used unobstructed cats (Osborne et al., 1996) and as such, these studies are not necessarily directly comparable. Two papers were also inaccessible for evaluation.

At present, there is insufficient evidence to prove absolutely whether meloxicam or prednisolone is the superior therapeutic agent for the treatment of FIC with respect to the reduction of clinical signs. The fact that two papers were also unavailable for assessment may also make these findings spurious, as these may offer strong evidence which answers the PICO question. Further studies comparing the efficacy of meloxicam and prednisolone as treatments for FIC may be required in order to answer the PICO question. Finally, it would be worth broadening the horizon of this question to include other commonly used and available therapies for treating FIC, since one of these treatments may prove effective where meloxicam and prednisolone may not.

## Methodology

**Search Strategy:** 

Databases searched and dates covered:	CAB Abstracts on Ovid Platform; 1973–2021 Week 14. PubMed on NCBI interface; 1920–April 2021.
Search strategy:	CAB Abstracts: (cat or cats or feline* or felid* or queen* or tom* or DSH* or DLH* or (domestic long?hair* or short?hair*))).mp. ((feline and (interstitial or idiopathic) and cystitis) or ((interstitial or idiopathic or sterile) and cystitis) or ((pandora or feline urologic) and syndrome*) or (FIC not "fractional inhibitory concentration") or FUS or FLUTD or "feline lower urinary tract disease").mp. (melox* or "loxicom" or "metacam" or "inflacam" or "rheumocam" or NSAID* or non?steroidal anti?inflammatory drug* or non?steroidal nati?inflammator* or anti?inflammator*).mp. (steroid* or glucocorticoid* or corticosteroid* or predni* or "prednicortone" or "prednicare" or prednisolone* or anti?inflammator*).mp. (resol* or reduc* or improve* or recover* or symptom* or treat* or therap* or clinical sign* or haematuria* or hematuria* or dysuria* or stranguria* or pollakiuria* or periuria* or void* or bladder inflammation or inappropriate* void* or voids inappropriate*).mp. 1 and 2 and 3 and 4 and 5 (9) 1 and 2 and 3 and 4 (9) 1 and 2 and 3 (12) 1 and 2 and 4 (17) 1 and 2 and 3 and 5 (12) 1 and 2 and 4 and 5 (16) 6 or 7 or 8 or 9 or 10 or 11 (20) PubMed: (cat or cats or feline or felid or queen or tom or DSH or DLH or (domestic and (long hair or longhair or short hair or shorthair))) feline interstitial cystitis or feline idiopathic cystitis or pandora urologic syndrome or feline urologic syndrome or FIC or FUS or FLUTD or feline lower urinary tract disease meloxicam or loxicom or metacam or inflacam or rheumocam or orocam or NSAID or non-steroidal anti-inflammatory drug or non-steroidal anti-inflammatory or anti inflammatory steroid or glucocorticoid or corticosteroid or prednisone or prednicortone or prednicare or prednisolone or anti inflammatory 1 and 2 and 3 and 4 (22)
Dates searches performed:	12 Apr 2021

**Exclusion / Inclusion criteria:** 

Exclusion:	Non-English language, popular press articles, conference abstracts, book chapters, opinion articles, conference papers.
Inclusion:	Studies comparing the time to resolution or improvement of clinical signs in cats with FIC when comparing meloxicam or prednisolone. Studies examining the efficacy of meloxicam in reducing the clinical signs of cats with FIC when compared to another treatment or placebo. Studies examining the efficacy of prednisolone in reducing the clinical signs of cats with FIC when compared to another treatment or placebo.

**Search outcome:** 

Database	Number of results	Excluded – Duplicates	Excluded – Non-English	Excluded – Conference paper and/or book chapter	Excluded – Entire journal issue*	Excluded – Did not answer PICO question	Total relevant papers
CAB Abstracts	20	0	1	6	1	10	2
Medline	22	5	0	0	0	17	0
Total relevant papers when duplicates removed							2

## Conflict of Interest

The authors declare no conflicts of interest.

The author would like to extend thanks to Bridget Sheppard, Clare Boulton and the remainder of the staff at RCVS Knowledge for their guidance throughout the process of writing the Knowledge Summary and support with the literature searches, as well as Sarah O'Shaughnessy at the University of Bristol for informing them of the ability to submit Knowledge Summaries through the *Veterinary Evidence* website.

## Disclaimer

Knowledge Summaries are a peer-reviewed article type which aims to answer a clinical question based on the best available current evidence. It does not override the responsibility of the practitioner. Informed decisions should be made by considering such factors as individual clinical expertise and judgement along with patient’s circumstances and owners’ values. Knowledge Summaries are a resource to help inform and any opinions expressed within the Knowledge Summaries are the author’s own and do not necessarily reflect the view of the RCVS Knowledge. Authors are responsible for the accuracy of the content. While the Editor and Publisher believe that all content herein are in accord with current recommendations and practice at the time of publication, they accept no legal responsibility for any errors or omissions, and make no warranty, express or implied, with respect to material contained within. For further information please refer to our Terms of Use.
